# The role of O-polysaccharide chain and complement resistance of *Escherichia coli* in mammary virulence

**DOI:** 10.1186/s13567-020-00804-x

**Published:** 2020-06-15

**Authors:** Hagit Salamon, Einat Nissim-Eliraz, Oded Ardronai, Israel Nissan, Nahum Y. Shpigel

**Affiliations:** The Koret School of Veterinary Medicine, Hebrew University of Jerusalem, POB 12, 76100 Rehovot, Israel

## Abstract

Mastitis, inflammation of the mammary gland, is a common disease of dairy animals. The disease is caused by bacterial infection ascending through the teat canal and mammary pathogenic *Escherichia coli* (MPEC) are common etiology. In the first phase of infection, virulence mechanisms, designated as niche factors, enable MPEC bacteria to resist innate antimicrobial mechanisms, replicate in milk, and to colonize the mammary gland. Next, massive replication of colonizing bacteria culminates in a large biomass of microbe-associated molecular patterns (MAMPs) recognized by pattern recognition receptors (PRRs) such as toll-like receptors (TLRs) mediating inflammatory signaling in mammary alveolar epithelial cells (MAEs) and macrophages. Bacterial lipopolysaccharides (LPSs), the prototypical class of MAMPs are sufficient to elicit mammary inflammation mediated by TLR4 signaling and activation of nuclear factor kB (NF-kB), the master regulator of inflammation. Using in vivo mastitis model, in low and high complements mice, and in vitro NF-kB luminescence reporter system in MAEs, we have found that the smooth configuration of LPS O-polysaccharides in MPEC enables the colonizing organisms to evade the host immune response by reducing inflammatory response and conferring resistance to complement. Screening a collection of MPEC field strains, we also found that all strains were complement resistant and 94% (45/48) were smooth. These results indicate that the structure of LPS O-polysaccharides chain is important for the pathogenesis of MPEC mastitis and provides protection against complement-mediated killing. Furthermore, we demonstrate a role for complement, a key component of innate immunity, in host-microbe interactions of the mammary gland.

## Introduction

Mastitis, inflammation of the mammary gland, is an important disease in dairy animals and *Escherichia coli* (*E. coli*) is a common etiology [1, 2]. Mammary infection is ascending through the teat canal and a small challenge of mammary pathogenic *E. coli* (MPEC) is sufficient to replicate in the milk spaces and colonize the gland. In the first phase of infection, virulence mechanisms, designated as niche factors, enable MPEC bacteria to resist innate antimicrobial mechanisms, replicate in milk, and to colonize the mammary gland [3]. Next, massive replication of colonizing bacteria culminates in a large biomass of microbial associated molecular patterns (MAMPs) recognized by pattern recognition receptors (PRRs) such as toll-like receptors (TLRs) mediating inflammatory signaling in mammary alveolar epithelial cells (MAEs) and macrophages [1, 4]. Bacterial lipopolysaccharides (LPS), the prototypical class of MAMPs are sufficient to elicit mammary inflammation mediated by TLR4 signaling and activation of nuclear factor kB (NF-kB), the master regulator of inflammation.

LPS is an important component of the outer membrane of gram-negative bacteria, contributing to the structural integrity of the bacterial cell wall and conferring resistance to chemical attack [5]. LPS consists of three distinct domains; the membrane-embedded lipid A, core oligosaccharides, and the O-antigen polysaccharides. LPS molecules that comprise all three regions are called smooth, while LPS molecules lacking the O-antigen are named rough. The lipid A moiety represents the active, proinflammatory moiety of LPS molecules as it binds directly to TLR4 [6]. Whereas the lipid A structure is relatively conserved, there is great variability in the composition of the O-polysaccharides between bacterial strains, which provides the major basis for bacterial serotyping. The O-polysaccharides chains are composed of four-sugar repeat units that externally protrude from the bacterial membrane [7], and the effects of their structural variability on the proinflammatory activity of LPS are less well studied [8, 9]. Other virulence traits that are not related to the proinflammatory activity of LPS might be affected by the length and structure of the O-polysaccharides, most notable are resistance to complement and phagocytosis and killing by macrophages and neutrophils [10–13]. The variable structure and antigenicity of the O-polysaccharides chains have been extensively used for classical serological O-typing of many gram-negative bacteria including MPEC. Thereupon many specific O-serotypes were linked to virulence, pathotypes and clinical syndromes, most notable examples are O157 enterohemorrhagic *E. coli* (EHEC), O127 enteropathogenic *E. coli* (EPEC). This simple technique has been extensively used for decades to classify MPEC isolates, however, specific O-types could not be linked to mammary virulence or clinical and epidemiological characteristics of the disease. Noteworthy, most isolates were serum resistant in all tested sera and serum resistance was the only characteristic that could be related to mammary virulence [14]. Furthermore, serum resistance and smooth colony morphology have been linked to virulence in many bacterial pathogens including MPEC [8, 15].

Using in vivo murine mastitis model and in vitro NF-kB luminescence reporter system in MAECs, we have found that the smooth configuration of LPS O-polysaccharides in MPEC enables the colonizing organisms to evade the host immune response by reducing inflammatory response and conferring resistance to complement. These results indicate that the structure of LPS O-polysaccharides chain is important for the pathogenesis of MPEC mastitis and provides protection against complement-mediated killing. Moreover, we demonstrate a role for complement, a key component of innate immunity, in host-microbe interactions in the mammary gland.

## Materials and methods

### Bacterial strains and plasmids

*E. coli* strain P4-NR (serotype O15:H21H54) was isolated from the milk of experimentally infected dairy cow [16–18]. Strain P4-96 is a variant of P4-NR derived by subcultures in our laboratory and is extensively described in the results. Strain P4 (serotype O32:H37) is a prototypical *E. coli* mastitis strain (unrelated to P4-NR and P4-96 strains) isolated from a bovine case of clinical mastitis [15]. Bacteria were transformed with plasmid pKB4985 which is based on pACYC184 constitutively expressing mCherry (low levels) [19] or with the plasmid pCP38 [20] constitutively expressing the *luxCDABE* operon of Photorhabdus luminescens, plasmids also include chloramphenicol and ampicillin resistance genes, respectively. Both plasmids were kindly donated by Prof. Ilan Rosenshine, Hebrew University, Israel. To select for mutant strains or carriage of plasmids, growth medium was supplemented with kanamycin (50 µg/mL), chloramphenicol (10 µg/mL), or ampicillin (100 µg/mL) as appropriate.

Pulsed-field gel electrophoresis of strains P4-NR and P4-96 was performed by Dr Lea Valinsky, (Ministry of Health Central Laboratories, Jerusalem, Israel), as previously described [21].

MPEC field strains were isolated by the National Service for Udder Health and Milk Quality (NSUHMQ) laboratory over 1 year. Quarter milk samples from dairy cows affected by clinical mastitis were originated from Israeli dairy herds, submitted to NSUHMQ laboratory and bacteriological examination was performed according to National Mastitis Council guidelines [22]. Strains were kindly donated by Dr. Mor Freed, NSUHMQ laboratory. All strains were cryopreserved in 20% glycerol at −80 °C until further processing.

### Construction of P4-NR and P4 mutants

The P4-NR∆*galU*::kan, P4-NR∆*galE*::kan, P4∆*galU*::kan, P4∆*galE*::kan mutants were obtained by P1_vir_ bacteriophage transduction from BW25113 (Keio Collection strain background) as previously described [23].

### Acriflavine agglutination test

**Figure 1:**
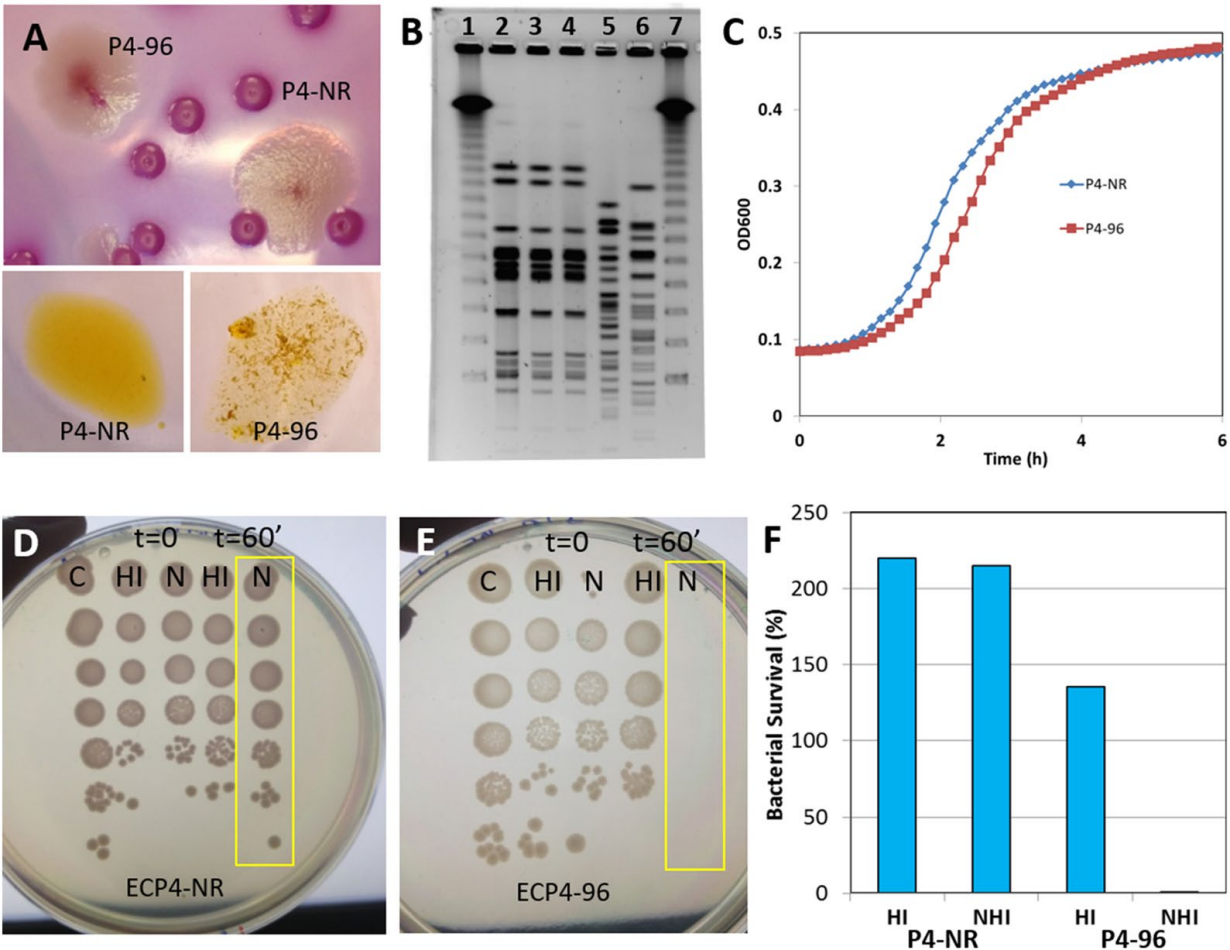
**Phenotypic and genotypic characteristics of MPEC strain P4-NR O15:H21H54 and its derivative P4-96.** P4-96 has rough colonies (top panel in **A**) and forms clumps upon mixing with acriflavine (lower panel in **A**), as opposed to P4-NR that has smooth colonies and does not aggregate. These strains are closely related genetically as demonstrated by PFGE (lanes 1-7 in **B**; 1-Lambda, 2-P4-96, 3-P4-NR, 4-P4-NR, 5-P333463, 6-K12, 7-Lambda). P4-96 displays reduced proliferation ability in vitro in comparison to ECP4-NR, as indicated by a comparative growth curve (**C**). P4-NR is serum resistant growing well in normal (N in **D**) and heat-inactivated (HI in **D**) bovine serum. P4-96 lack resistance to bovine serum, as indicated by no growth after 60 min of incubation with 80% normal bovine serum (N in image **E**) and growth in heat-inactivated bovine serum (HI in **E**). Growth in LB broth were used as controls (C in **D**–**E**). Bacterial growth in heat-inactivated (HI) or non-HI (NHI) was also quantified and presented in (**F**). Figures are of representative results from 3 or more similar experiments.

Colony morphology was visually examined and photographed after incubation at 37 °C for 48 h on LB or MacConkey agar plates (Figure 1). The acriflavine agglutination test (AAT) was used to distinguish between smooth and rough strains [24]. We developed and validated two AATs; slide AAT and plate AAT. Approximately 10 μL of a 0.1% (wt/vol) aqueous solution of acriflavine (Sigma, Israel) was mixed with 10 μL of bacterial cell suspension (~10^10^ cells/mL) on glass slides or in 96-well plates. The absence of agglutination indicates a smooth culture while agglutination was clearly visible with rough strains (Figures 1, 4, Additional file 3).

### Serum sensitivity test

Blood taken from healthy dairy cows was left to clot at 37 °C for 45 min, and the serum was separated from the clot by centrifugation (10 min, 700 × *g*). Aliquots containing 0.2 mL (LB broth) of bacterial culture in early log phase (OD600 of 0.2) were mixed with 0.6 mL serum and incubated at 37 °C. Samples of 0.1 mL were diluted and plated for viable count at different time points. Complement was inactivated by incubating the serum for 20 min at 56 °C before mixing it with the bacterial culture.

### *Galleria mellonella* infection model

Considering the high severity of systemic bacterial sepsis studies in mice, we have used infection of wax moth larvae as an adequate replacement model system for the study of in vivo virulence [25]. *Galleria mellonella* larvae (kindly donated by Dr Itamar Glazer, ARO, Volcani Center, Israel) were grown on artificial diet as previously described [26]. *G. mellonella* larvae were injected via the hindmost left proleg with 10 µL of bacterial suspension (the inoculum size was as described in the results) and were incubated at 37 °C in the dark. Larvae were individually examined for pigmentation, and time of death was recorded. For the assessment of CFUs, larvae were decontaminated by 70% ethanol, and homogenized by a hand-held homogenizer in a 50-mL conical tube. Serial dilutions were plated to assess CFU number.

### Murine mastitis model

Six- to eight-week-old female C57BL/6 and C3H/HeN mice were used in this study (Envigo, Jerusalem, Israel). All mice were maintained under specific pathogen-free conditions and handled under protocols approved by the Hebrew University Animal Care Committee, according to international guidelines. IACUC approvals were obtained prospectively (Ethics Committee for Animal Experimentation, Hebrew University of Jerusalem; MD-13-13704-3 and MD-16-14874-3). Bacterial intramammary challenge was performed 7–10 days post-partum. Mice were challenged by intramammary (IMM) infusion through the teat canal, as previously described [18]. Decomplementation of mice was performed using cobra venom factor (CVF from naja naja kaouthia; ACZON S.p.A., Bologna, Italy) as previously described [27]. Mice were injected intraperitoneally with 50 μg CVF in PBS 24 h before IMM challenge.

### Preparation of challenge organism

Bacteria from frozen stocks were pre-cultured in shaker incubator for 5 h at 37 °C in Brain Heart Infusion Broth (BHI, Difco, Becton, Dickinson and Company, NJ, USA). Pre-cultured bacteria were diluted 1/100 with fresh BHI broth and incubated overnight at 37 °C under static conditions. Next, culture was centrifuged for 15 min at 4000 rpm and bacteria were similarly washed twice with sterile normal saline. Bacteria were resuspended and inoculum was prepared and quantified by plating serial 10-fold dilutions on agar plates. P4-NR and P4-96 were inoculated as single strains or co-inoculated at a 1:1 ratio in competition study and mammary tissues were harvested for bacterial counts 24 h after challenge. The competitive index was calculated by dividing the ratio of P4-96 (chloramphenicol resistant) population versus P4-NR population at the end of the infection by the ratio of P4-96 population versus P4-NR population in the initial inoculum.

### Bioluminescence and fluorescence imaging

In vivo imaging of IMM challenged animals was performed 24 h after infection. Hair was shaved and depilated (Nair hair removal cream) from the ventral side of the animals. Mice were injected intraperitoneally with 200 mg/kg luminol (Santa Cruz Biotechnology, TX, USA) 10 min before bioluminescence imaging of neutrophil recruitment [28]. The mice were anesthetized (1.5–2.5% isofluorane in O_2_) and a whole-body image was acquired using IVIS Lumina Series III (PerkinElmer Inc., MA, USA) with excitation/emission filters blocked/open for luminescence (luminol), 560/620 for mCherry fluorescence and 460/520 for milk autofluorescence. Next, the mice were sacrificed, the mammary glands were exposed, and imaging was repeated as described above. Living Image Software version 4.4 (Caliper LifeSciences, MA, USA) was used to display each image as a false-color photon-count image superimposed on a grayscale anatomic image.

For in vivo imaging of challenged wax moth larvae, 3 larvae were challenged with 10^4^ CFUs of strain P4-NR harboring the bioluminescence pCP38 plasmid. The challenged larvae were fitted into plastic tubes interspaced and plugged with cotton wool. Repeated images were acquired every 15 min over 9 h using IVIS Lumina with excitation/emission filters blocked/open for luminescence. ROI were drawn around each individual larva and counts in the ROI detected by CCD camera digitizer were converted to physical units of radiance in photons/s/cm^2^/steradian.

### Histological analysis and bacterial counts

Mice were killed 24 h after challenge and mammary tissues were trisected for histology, total RNA extraction and total bacterial counts as previously described [4, 29–31]. Samples for histological analysis were fixed in neutral buffered 4% paraformaldehyde (PFA), paraffin embedded, and sections were cut at a thickness of 5 µm and stained with hematoxylin and eosin (H&E).

### Real-time PCR

Quantitative real-time PCR (qPCR) was performed as previously described [32, 33]. Total RNA was isolated from mammary tissue using the GeneElute Mammalian Total RNA Miniprep Kit (Sigma, Rehovot, Israel) combined with on-Column DNase I Digestion Set (Sigma). Reverse transcription was performed using qScript cDNA Synthesis Kit (Quanta BioSciences, Gaithersburg, MD, USA) and cDNA was used for subsequent real-time PCR reactions. Quantitative real-time RT–PCR was conducted on a StepOne Plus PCR instrument (Applied Biosystems) using the FAST qPCR Universal Master Mix (Kappa Biosystems, Boston, MA, USA). PCR primers used in this study are listed in Additional file 1. All reactions were performed in triplicates and the gene expression levels for each amplicon were calculated using the ΔΔCT method [34] and normalized against HSP90 mRNA. Melting curve analysis was performed on each primer set to confirm amplification of a single product and all amplicons were sequenced to ensure reaction specificity (data not shown).

### NF-kB luminescence reporter system

To create lentiviral NF-kB reporter vector, a target plasmid (transfer vector) was constructed to express luminescence reporter in mouse mammary epithelial line EPH4. The luminescence reporter was constructed of destabilized firefly luciferase (pGL4.24-luc2P, Promega) [35, 36] open reading frame controlled by a DNA cassette containing five tandem repeats of the NF-k-B transcriptional response element (pLNV-minP-5kB-luc2P). The lentiviruses were produced from HEK293FT cells (ThermoFisher scientific, MA, USA) through co-transfecting the target plasmid pLNV and the packaging vectors pMDLg-RRE, pRSV-REV and pCMV-VSVG as previously described [37]. Co-transfection using VSVG plasmid encoding for envelope G glycoprotein from VSV, produced pseudotyped retrovirus which depends on the expression of LDLR on plasma membrane for the infection of target cells [38, 39]. Lentiviruses were purified by ultracentrifugation and then quantitated as previously described [37]. All plasmids were received from Dr Pawel Paszek, University of Manchester.

EPH4 cells were infected with the lentiviral NF-kB reporter vector and stably transduced cells were used. EPH4 cells were cultured in Dulbecco’s modified Eagle medium (DMEM) supplemented with 10% fetal calf serum (FCS) (Biological Industries, Beit HaEmek, Israel) and 100 units/mL penicillin, 0.1 mg/mL streptomycin (Pen-Strep; Biological Industries), 1% l-Glutamine (BI) and 1% HEPES buffer (BI) at 37 °C with 5% CO_2_. Cells were seeded onto 24-well plates at a density of 3 × 10^5^ cells/well in 500 µL of culture medium (without antibiotics) 24 h prior to infection with MPEC strains described in the results. Bacteria were grown in Luria–Bertani (LB) broth at 27 °C. For in vitro challenge studies, bacterial cultures were diluted in cell culture medium to multiplicity of infection (MOI) of 1. Normal medium and medium containing 1 µg/mL LPS (from *E. coli* serotype O55:B5, Sigma, Rehovot, Israel) were used as negative and positive controls, respectively. Cells were plated at equal density and luminescence signals were quantified in the presence of 150 µg/mL D-luciferin (GoldBio) using SpectraMax i3x multiple detection microplate reader (Molecular Devices, CA, USA).

### Statistics

Median CFU counts and competition index were calculated at 24 h after challenge. Experimental groups were compared by the non-parametric Mann–Whitney two-independent-samples test. Competition index values were first subjected to a square root transformation, the values did not follow a normal distribution, and therefore, non-parametric statistics were used. CI was analyzed using One-Sample Wilcoxon Signed Rank Test and the null hypothesis was that median of CI equals 1. Survival analysis of wax moth larvae following bacterial challenge was performed using the Log-rank (Mantel-Cox) test.

All statistical analyses were performed using GraphPad Prism 6 (GraphPad Software, Inc., CA, USA) and a *P* value of 0.05 or less was considered significant.

Relative expression of cytokines, chemokines and Ly6G transcripts was analyzed by cluster analysis using Non-metric multidimensional scaling (NMDS) tested with Bray–Curtis similarity index. 95% concentration ellipses were plotted in scatter plots for NMDS. Shepard plot was obtained versus observed (target) ranks and indicates the quality of the result. Ideally, all points should be placed on a straight ascending line (x = y). The R^2^ values are the coefficients of determination between distances along each ordination axis and the original distances. Cluster analysis was performed using PAST3 software version 3.04.

## Results

### Characterization of MPEC P4-NR O15:H21H54 in the murine mastitis model

*E. coli* strain P4-NR O15:H21H54 was previously isolated from a case of bovine experimental mastitis [16]. Repeated subcultures of the parent strain lead to the appearance of a colony morphology variant strain (Figure 1A), designated as strain P4-96. Pulsed-field gel electrophoresis (PFGE) analysis of P4-NR and P4-96 demonstrated that these 2 strains are genetically closely related (Figure 1B). The two strains were comparatively analyzed for growth in LB broth (Figure 1C), acriflavine agglutination test (Figure 1A; bottom panel), resistance to normal (N) and heat-inactivated (HI) bovine serum (Figures 1D–F), and systemic virulence in wax moth larvae (Additional file 2). In summary, the emerging rough strain P4-96 was closely related to the parent strain P4-NR and lost serum resistance and in vivo systemic virulence in wax moth larvae attributed to the parent strain.

**Figure 2:**
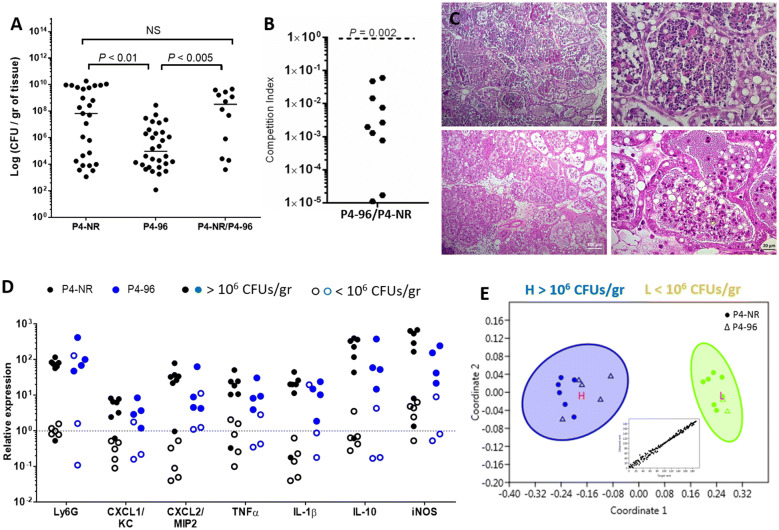
**Characterization of MPEC P4-NR O15:H21H54 and sub strain P4-96 in the murine mastitis model.** Mammary virulence of P4-NR and attenuation of P4-96 are demonstrated in lactating C57BL/6 mice following IMM challenge of L4 and R4 glands with ~1000 CFUs of bacteria. P4-NR and P4-96 were inoculated as single strains or co-inoculated at a 1:1 ration in competition study. 24 h after challenge mammary tissues were harvested for bacterial counts (scatter plot in **A**), each data point represents a single gland, and the horizontal bars indicate the median of data from three or more independent experiments. Mammary colonization by P4-96 was significantly reduced in C57BL/6 (**A**) mice and reduced fitness of strain P4-96 is demonstrated after co-challenge studies in C57BL/6 mice where P4-NR outcompeting P4-96 in the mammary gland (**B**), competition index significantly < 1. Massive neutrophil recruitment into alveolar milk space is demonstrated in representative microscope images of H&E stained paraffin sections (P4-NR; top panels in **C**, P4-96; lower panels in **C**). Neutrophil recruitment was also quantified by measuring the relative expression of Ly6G gene in mammary tissues using qPCR (black markers for P4-NR and blue markers for P-96 in **D**). Inflammation was further quantified by measuring the relative expression of the following genes in mammary tissues; KC, MIP2, TNFα, IL-1β, IL-10 and iNOS (**D**). Glands with high bacterial counts (> 10^6^ CFUs/gr tissue; filled markers in **D**) show consistently higher expression of inflammatory markers than glands with low counts (< 10^6^ CFUs/gr tissue; open markers). Relationships between bacterial counts and inflammatory variables, which include cytokines, chemokines and Ly6G, were further visualized using non-metric multidimensional scaling (**E**). Scale bars; 100 µm (left panels in **C**) and 20 µm (right panels in **C**).

Taken together, we concluded that strain P4-96 was highly attenuated and thus formulated our next aim to compare the two strains as mammary pathogens. To this end, lactating C57BL/6 mice were challenged with ~10^3^ CFUs of P4-NR and P4-96 and mammary infection and inflammation were characterized 24 h post-infection by mammary bacterial counts (Figures 2A and B), histological analysis (Figure 2C) and expression levels of inflammatory indicators (Figure 2D). Diseased glands were characterized by high bacterial counts (> 10^6^ CFUs per gram of tissue) and perfuse neutrophil recruitment into milk spaces which was also quantified using qPCR to measure the relative expression of the LY6G gene (left column in Figure 2D). Moreover, the relative expression of the genes MIP2, KC, IL10, IL1β, TNFα, and iNOS were also increased in these glands (Figure 2D). Using cluster analysis, glands were classified as high (> 10^6^ CFUs/gr) and low (< 10^6^ CFUs/gr) bacterial counts and were analyzed against all inflammatory markers measured in each gland. Interestingly, inflammatory markers clustered, irrespective of challenge strain, with distinction between bacterial biomasses (Figure 2E). This level of bacterial biomass defines the cutoff of bacterial growth required for the activation of mammary inflammation as was previously suggested [40].

These results clearly established P4-NR and P4-96 as MPEC strains in lactating C57BL/6 mice.

### P4-96 strain is attenuated in mammary colonization, fitness and virulence

Although both strains were established as mammary pathogens in lactating C57BL/6 mice, mammary colonization with strain P4-96 was significantly reduced compared to strain P4-NR (Figure 2A). Moreover, in co-challenge study (total counts in right column of 2A), strain P4-NR significantly outcompeted strain P4-96 (Figure 2B) clearly demonstrating reduced mammary fitness of P4-96 in comparison with the parent strain.

**Figure 3:**
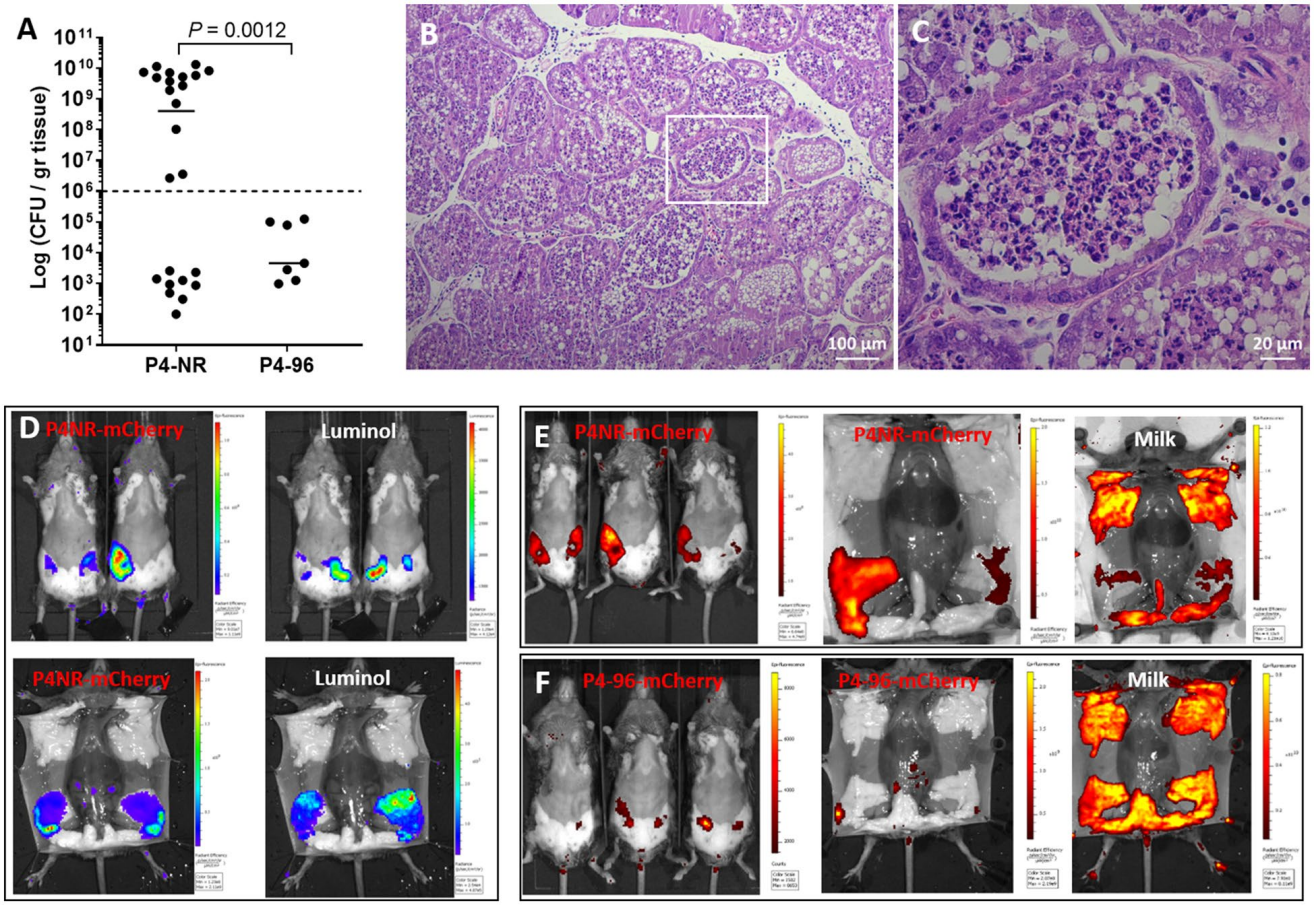
**MPEC P4-96 strain is attenuated in mammary colonization, fitness and virulence.** Mammary virulence of P4-NR and attenuation of P4-96 were demonstrated in lactating C3H/HeN mice 24 h after IMM challenge of L4 and R4 glands with 10^4^ CFUs of bacteria. 24 h after challenge mammary tissues were harvested for bacterial counts (scatter plot in **A**), each data point represents a single gland, and the horizontal bars indicate the median of data from three or more independent experiments. Statistical significance was determined by non-parametric Mann–Whitney two-independent-samples test (**A**) using GraphPad Prism 6 (GraphPad Software, Inc.) and *P* value of 0.05 or less was considered significant. Mammary colonization by P4-96 was significantly reduced in C3H/HeN mice (**A**). Massive neutrophil recruitment into alveolar milk spaces was only elicited following challenge with P4-NR strain and is demonstrated in representative microscope images of H&E stained paraffin sections (**B**–**C**). Attenuated disease was also demonstrated using whole body imaging 24 h after IMM challenge with 10^4^ CFUs P4-NR (**D**–**E**) or P4-96 (**F**) bacteria expressing the fluorescent reporter mCherry. Fluorescence and bioluminescence imaging was performed using IVIS Lumina Series III (PerkinElmer Inc., MA, USA) in live mice (**D**; top panels and **E**; left panels) and of exposed glands in euthanized mice (**D**; bottom panels and **E**; middle and right panels). Each image is displayed as a false-color photon-count image superimposed on a grayscale anatomic image. Mammary colonization by P4-NR is widespread and the fluorescence signal is correlated with the high bacterial counts in the mammary tissue (**D**; left panels and **E**; left and middle images). Mammary virulence of P4-NR was correlated with neutrophil recruitment to the mammary glands quantified by bioluminescence imaging of myeloperoxidase (MPO) upon systemic administration of luminol (**D**; right panels) and visible decrease in milk production in challenged glands (**E**; right bottom panel). P4-96 attenuation is demonstrated by focal colonization (**F**; left and middle images) and no visible decrease in milk production (**F**; right image). Representative images of ≥ 6 mice challenged by each bacterial strain. Scale bars; 100 µm (**B**) and 20 µm (**C**).

These differences between P4-NR and P4-96 strains were further enhanced following challenge studies in C3H/HeN mice. In this mouse strain, initial attempts with IMM challenge dose of ~1000 CFUs of strain P4-96 failed (data not shown) and the challenge dose was increased to ~10 000 CFUs per gland. Using this higher dose, strain P4-96 was able to colonize the glands albeit achieving significantly lower counts than its parent strain P4-NR (Figure 3A). Histological analysis of the infected areas of the gland revealed the characteristic hallmarks of the disease as described above (Figures 3B, C). Next, we used fluorescence and bioluminescence whole body imaging to visualize the spatial distribution of colonizing bacteria expressing the fluorescent protein mCherry, neutrophil recruitment (luminol bioluminescence) and milk production (autofluorescence) in the mammary gland (Figures 3D, E). Neutrophil recruitment to the mammary glands was quantified by bioluminescence imaging of myeloperoxidase (MPO) upon systemic administration of luminol. Challenge with the parent strain P4-NR resulted in diffuse bacterial colonization, neutrophil recruitment and reduced milk production in affected glands (Figures 3D, E). Loss of virulence by the P4-96 strain was further demonstrated by focal colonization which was limited to the teat areas, lack of luminescence signaling and unaffected milk production (Figure 3F).

**Figure 4:**
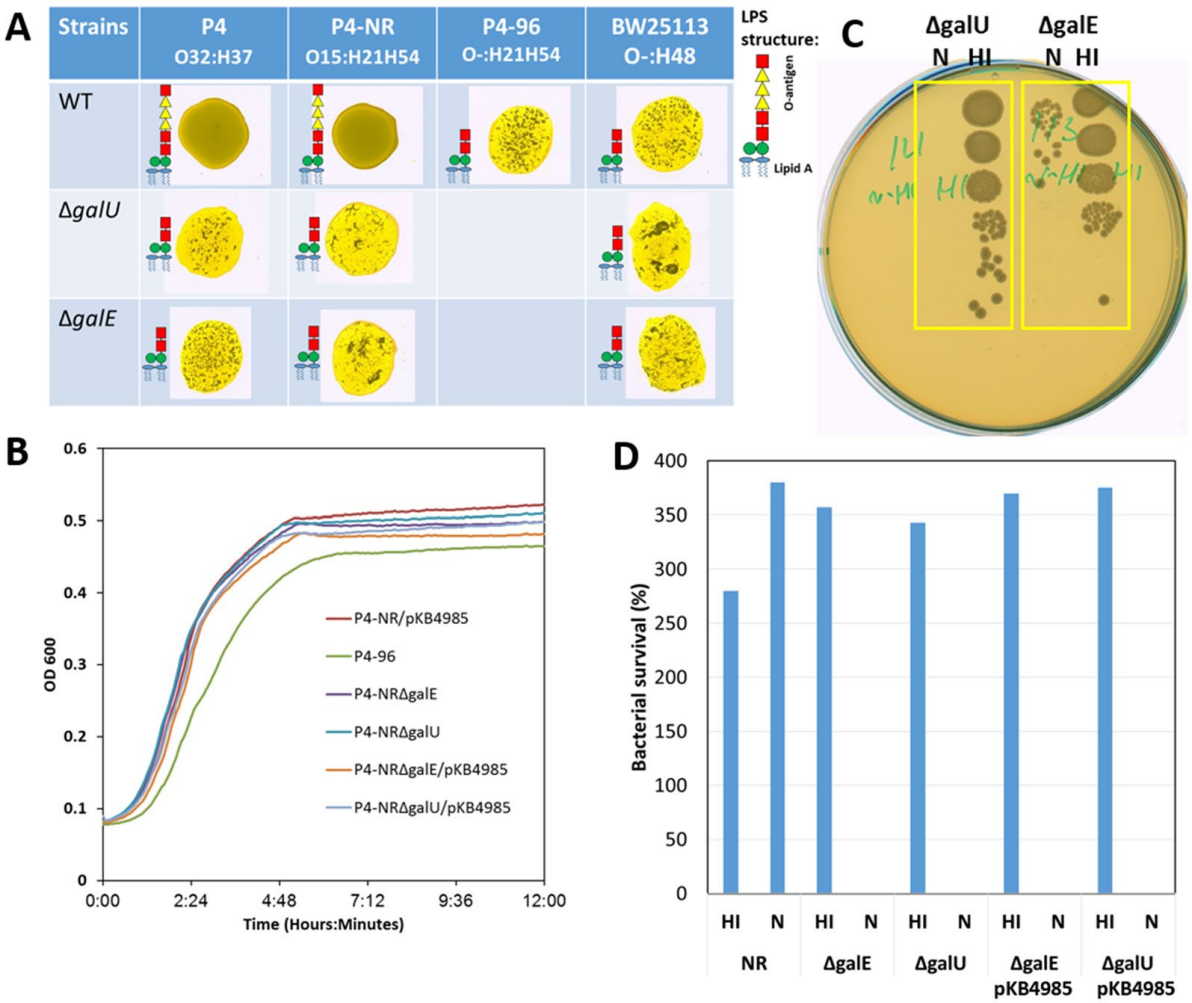
**P4-NR∆*galU*and P4-NR∆*galE*are rough mutants of P4-NR WT strain.** Phage transduction was used to transfer the mutation from *E. coli* BW2511 mutants in the same genes. The rough mutants form clumps upon mixing with acriflavine (**A**), as opposed to P4-NR that has smooth colonies and does not aggregate. The rough phenotype is attributed to loss of O-antigen chain of bacterial LPS while wild type strain P4-NR with full length O-antigen is smooth (**A**). All strains show similar growth in LB broth including those expressing the fluorescent protein mCherry (pKB4985 in **B**). The rough mutants lack resistance to bovine serum, as indicated by no growth after 60 min of incubation with 80% normal bovine serum (N in **C**) and growth in heat-inactivated bovine serum (HI in **C**). Bacterial growth in heat-inactivated (**D**; HI) or normal (**D**; N) serum was also quantified and presented in **D**. Figures are of representative results from 3 or more similar experiments. P4-NR is serum resistant growing well in normal (**C**; N in D) and heat-inactivated (HI in D) bovine serum.

Taken together we show here that the rough and serum sensitive variant P4-96 was highly attenuated in mammary fitness and virulence. The rough phenotype is attributed to loss of O-polysaccharides (O-PS) chain of bacterial LPS while wild type strains with full length O-PS are termed smooth [41, 42]. The O-PS chains are known to be involved in complement resistance since smooth-type bacteria are serum resistant. We hypothesized that “smooth” O-PS chain structure and serum resistance are important virulence attributes of MPEC bacteria. Accordingly, we produced targeted *galU* and *galE* mutants [43] of P4-NR rendering these strains rough and serum sensitive (Figure 4). The mutant strains were evaluated for mammary colonization, fitness and virulence in comparison with wild type strain.

### Loss of mammary virulence by P4-NR∆*galU* and P4-NR∆*galE*

**Figure 5:**
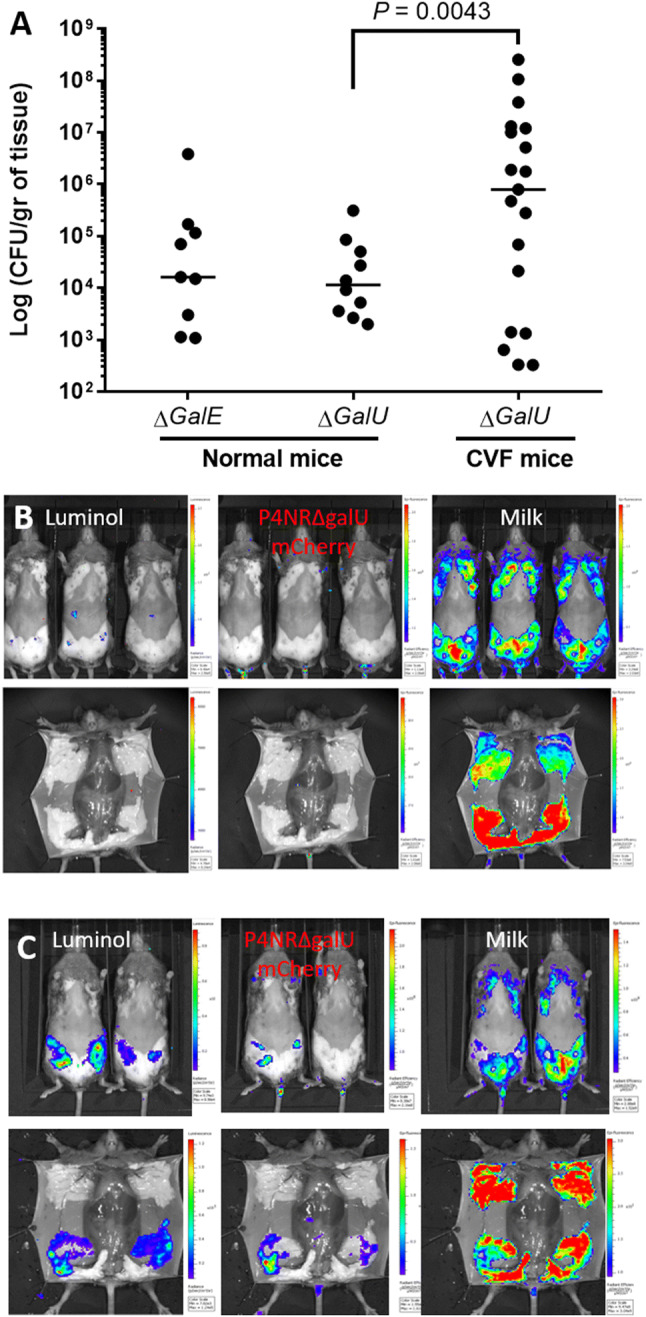
**Resistance to complement is an essential virulence trait in mammary pathogenic*E. coli***. Loss and rescue of mammary virulence were demonstrated in normal and decomplemented C3H/HeN mice, respectively. Normal mice (left and middle columns in **A** and panel **B**) or mice decomplemented by IP injection of cobra venom factor (CVF; right column in panel **A** and panel **C**) well challenged by IMM infusion of L4 and R4 glands with 10^4^ CFUs of complement-sensitive rough P4-NR∆*galU* and P4-NR∆*galE* strains. 24 h after challenge mammary tissues were harvested for bacterial counts (scatter plot in **A**), each data point represents a single gland, and the horizontal bars indicate the median of data from three or more independent experiments. Loss of virulence (**B**) and rescue (**C**) were also demonstrated using whole body imaging 24 h after challenge for bacterial growth (mCherry fluorescence; middle columns in **B** and **C**), neutrophil recruitment (luminol bioluminescence; left columns in **B** and **C**) and milk in the glands (autofluorescence; right columns in **B** and **C**). Fluorescence and bioluminescence imaging was performed using IVIS Lumina Series III (PerkinElmer Inc., MA, USA) in live mice (top panels in **B** and **C**) and of exposed glands in euthanized mice (bottom panels in **B** and **C**). Each image is displayed as a false-color photon-count image superimposed on a grayscale anatomic image. Bacterial growth, neutrophil recruitment and reduced milk were not visible following challenge with *galU* mutant strain (**B**). Mammary colonization (**A** and **C**) and virulence (**C**) of P4-NR∆*galU* bacteria were regained in decomplemented mice demonstrated by bacterial counts and whole body imaging. Representative images of ≥ 6 mice challenged by each bacterial strain.

Lactating C3H/HeN mice were challenged by IMM infusion of ~10 000 CFUs of P4-NR∆*galU* or P4-NR∆*galE* bacteria. These mutant strains were clearly colonization defective as demonstrated by low mammary bacterial counts 24 h after challenge (Figure 5A). Using whole body imaging, mice were also imaged 24 h after challenge for bacterial growth (mCherry fluorescence), neutrophil recruitment (luminol bioluminescence) and milk in the glands (autofluorescence) (Figure 5B). Bacterial growth, neutrophil recruitment and reduced milk were not visible following challenge with *galU* (Figure 5B) or *galE* (data not shown) mutant strains. We concluded that deletion of the *galU* or *galE* genes in P4-NR resulted in reduced mammary colonization and loss of virulence.

The complement system is known to be active in milk and plays an important role in the innate response against invading bacteria through its bactericidal activity [44] against myriad of organisms including serum sensitive *E. coli* [45]. Considerable differences are known to exist in complement activity between inbred mouse strains. Complement activity is high and normal in C3H/HeN mice, lower in female C57BL/6 mice and deficient in DBA/2 mice [46, 47]. These differences might explain the partial susceptibility of C57BL/6 mice and higher resistant of C3H/HeN mice to IMM challenge with rough strains (P4-NR∆*gal* and P4-96) which are also serum sensitive and complement susceptible. Hence, we hypothesize that complement depletion of C3H/HeN mice will render them susceptible to IMM challenge with the rough strain P4-NR∆*galU*.

### P4-NR∆*galU* regains mammary virulence in complement depleted mice

Decomplementation of mice was performed using cobra venom factor (CVF from *Naja naja kaouthia*; ACZON S.p.A., Bologna, Italy). CVF is an analogue of the complement protein C3b and prevents the formation of the terminal C component by binding factor B, thus forming a relatively stable C3/C5 convertase. This triggers the activation of the complement cascade in CVF-treated animals, resulting in the depletion of C3b and C5b from the circulation. Thus, CVF treatment induces a consumption of C3 and downstream components (C5–C9) [27]. Mice were injected intraperitoneally with 50 μg CVF in PBS 24 h before IMM challenge and decomplemented mice were challenged with 10 000 CFUs P4-NR∆*galU* bacteria by IMM infusion. Mammary colonization (Figures 5A, C) and virulence (Figure 5C) of P4-NR∆*galU* bacteria were regained in decomplemented mice demonstrated by bacterial counts and whole body imaging. Taken together our results suggest that bactericidal activity of milk complement inhibit mammary colonization by sensitive MPEC bacteria. For this reason, we hypothesized that most MPEC field strains should be smooth and complement resistant.

### MPEC field strains are smooth and serum resistant

A collection of clinically and epidemiologically defined MPEC field strains were analyzed in duplicate using the plate AAT (Additional file 3). The vast majority (45/48; 94%) were smooth and all were serum resistant. The rough, albeit serum resistant, MPEC strains might have evolved alternative evasion mechanisms to resist the bactericidal effect of complement [42].

### Rough MPEC strains are better activators of inflammatory response in mammary epithelial cells

Increased virulence of smooth gram negative bacteria is usually attributed to structural resistance of bacterial cell wall to the bactericidal effect of complement. However, steady state complement activity in normal milk is very low and most probably does not interfere with initial colonization and proliferation of invading MPEC organisms. In mastitis, complement activity in milk is rapidly increased to high levels which might affect the growth and viability of sensitive MPEC organisms. Thus, we compared the activation NF-kB, the master regulator of inflammation, in mammary epithelial cells by smooth and rough MPEC strains.

**Figure 6:**
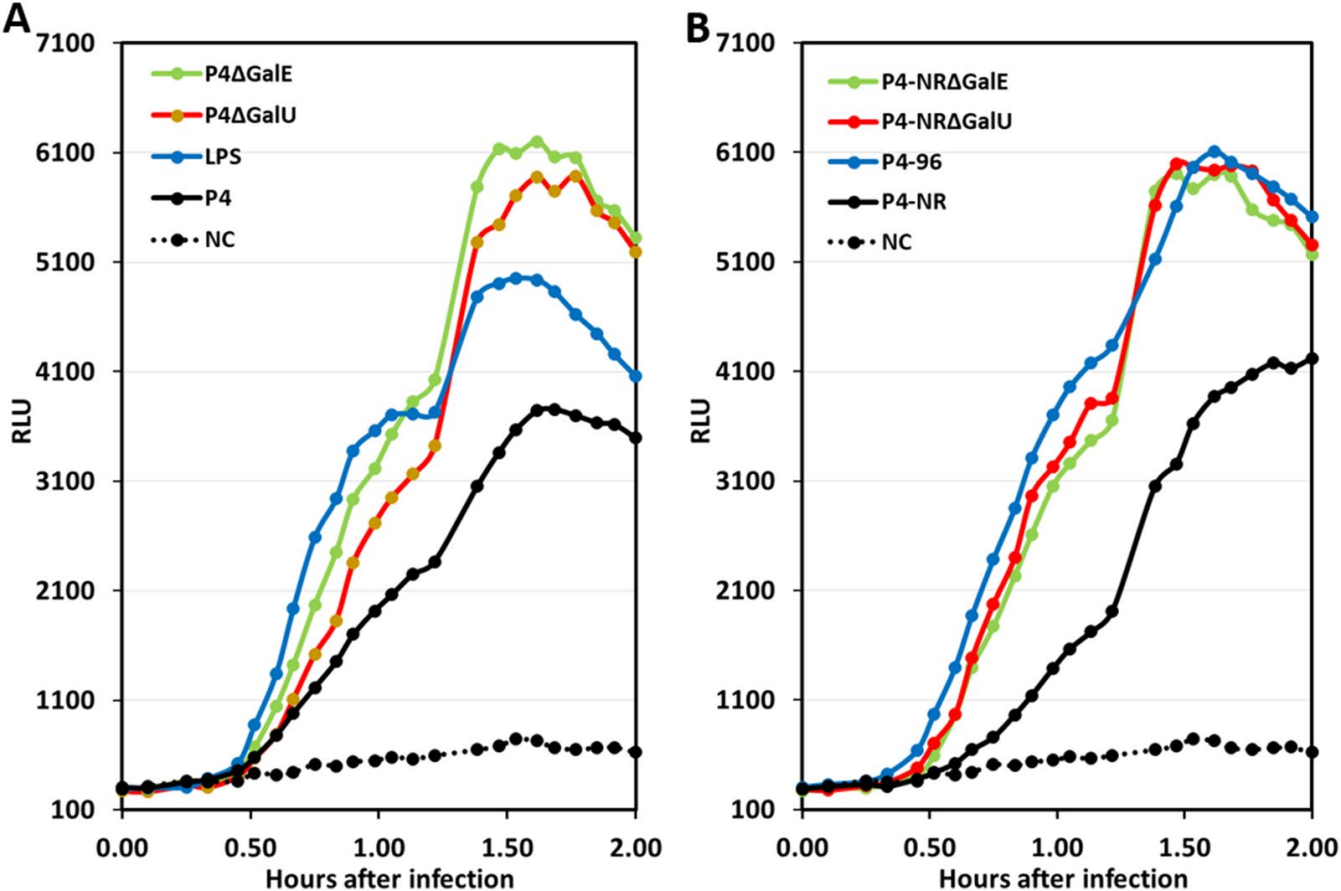
**Rough MPEC strains are better activators of inflammatory response in mammary epithelial cells.** Using lentivirus vector, mammary epithelial cells EPH4 were transduced with luciferase gene under the control of 5 copies of the NF-kappa-B response element. Transduced EPH4 cells were treated with 1 µgr/mL LPS (blue line **A**), and the following *E. coli* strains at MOI = 1; P4 (black line **A**), P4∆GalE (green line **A**), P4∆GalU (red line **A**), P4-NR (black line **B**), P4-96 (blue line **B**), P4-NR∆GalE (green line **B**), P4-NR∆GalU (red line **B**). NF-kappa-B activation was measured via analysis of luciferase activity in the presence of 150 µg/mL d-luciferin (RLU; relative luminescence units) following infection with the above described bacterial strains using SpectraMax i3x multiple detection microplate reader (Molecular Devices, CA, USA). In all experiments, LPS and normal medium were used as positive and negative controls, respectively. Data points are mean for one experiment performed in triplicate and represent the results of the three similar experiments.

EPH4 cells were infected with the lentiviral NF-kB luminescence reporter vector. Stably transduced cells were infected with MPEC strains P4, P4-NR, their *galU* and *galE* mutants (Figure 4) and rough field strains 26, 27 and 30 (Additional file 4). Infection by rough MPEC strains resulted in rapid and enhanced activation of NF-kB in mammary epithelial cells in comparison with smooth MPEC strains (Figure 6). Mammary alveolar epithelial cells (MAEC) are activated by microbial components to produce inflammatory mediators deranging the blood-milk barriers [48]. Blood is the major source of complement leaking through the perturbed blood-milk barrier attaining very high levels in milk [44, 49]. MAEC seem to be more sensitive to activation in response to rough MPEC strains which might explain earlier elimination and reduced fitness of these strains in the mammary gland.

## Discussion

*E. coli* strains causing extraintestinal infections are commonly designated as extraintestinal pathogenic *E. coli* (ExPEC) [50]. Much like other ExPEC, the most probable source of all mammary pathogenic *E. coli* (MPEC) bacteria is animal feces, these bacteria are resident or transient non-pathogenic gut commensals and constitute a small component of the normal intestinal microbiota. The diversity of *E. coli* strains in the gut of dairy cows is currently unknown and the relationships between gut commensalism and mammary virulence probably reflect some local adaptation to commensal habitats rather than virulence per se [51]. Our results suggest that all MPEC strains are complement resistant and that the complement system plays a key role in mammary response to invading MPEC bacteria. In the gut, mucosal epithelial cells highly express and secrete various complement components in response to MAMPs produced by the normal luminal microbiota [52, 53]. Commensal *E. coli* bacteria which are known to populate the mucus layer of the gut are exposed to the bactericidal activity of the complement system. This selective pressure in the habitats of gut commensal *E. coli* strains may coincidentally promote the emergence of virulence factors, rendering commensal *E. coli* strains reservoirs of mammary virulent strains.

Likewise, genes coding for iron capture systems have been correlated with successful gut colonization in humans and animals [51] and might explain their essential role in mammary colonization as recently suggested [3, 54]. Once again, this is an argument for the idea that these genes were at the origin selected and then maintained for giving a better fitness to strains within the intestine, the normal habitat of *E. coli*, and that their role in mammary virulence is “coincidental” [17, 55]. Nevertheless, much like other ExPEC, some studies might indicate that not all gut commensal *E. coli* are equally adapted to become MPEC [56].

The complement system is part of the defense against invading pathogens, with an essential role in both innate and adaptive immunity [44]. It is composed of more than 40 plasma and membrane proteins. It can be activated via three distinct routes: the classical (antibody dependent), lectin, and alternative pathways. Activation of complement cascades leads to the formation of the key component C3b on the bacterial surface, which stimulates phagocytosis. Late complement components (C5 to C9 proteins) are also activated via C3b, resulting in the formation of the membrane attack complex (MAC) causing bacterial cell lysis. The primary source of complement is blood, but complement proteins are also synthesized by a variety of other cell types and tissues including mammary epithelial cells [57]. Complement are present in milk of healthy uninflamed glands at low but significant concentrations. However, the classical pathway is not functional in milk due to the lack of C1q, but the alternative pathway is activated following bacterial infection leading to the formation of MAC and the pro-inflammatory fragment C5a [49]. Moreover, complement were reported to be produced by human keratinocytes [58, 59], and might also be produced by teat canal keratinocytes that may affect initial colonization by mammary pathogens.

The importance of complement activity was investigated in this study using the murine mastitis model system in line bred mice with low (C57BL/6) and high complement activity (C3H/HeN), and in complement-depleted mice. Low complement mice demonstrated increased susceptibility to rough MPEC strains which are not mammary pathogenic in high complement mice. Screening an epidemiologically defined collection of MPEC strains isolated from clinical cases of mastitis in dairy cows we found that most of these were smooth (94%) and all were complement resistant. Taken together, these observations suggested mammary fitness of *E. coli* bacteria is dependent on resistance to complement conferred mostly by smooth configuration of LPS. Using in vitro cell system, we also found that rough strains are stronger activators than smooth strains of NF-kB in mammary epithelial cells. NF-kB is a central component of the cellular signaling machinery that regulates inflammation and immunity in all body tissues including the mammary gland [60]. Nuclear transport of activated Rel/NF-kB proteins and binding to specific DNA binding sites transcriptionally upregulate the expression of > 200 NF-kB-dependent genes including complement genes. In mammary cells, NF-kB proteins are activated by signaling pathways downstream to microbial recognition receptors and cytokines receptors [61]. In the udder, steady state complement activity is considerably augmented following activation of NF-kB by replicating bacteria which is mediated by increased expression of complement in mammary cells and due to inflammation-induced leakage of milk-blood barrier. Furthermore, activation of NF-kB by complement components was previously described [62], whether this is also the case in mastitis still needs to be addressed. Our mechanistic model suggests that mammary fitness and virulence of smooth MPEC strains depend on their capacity to evade steady state and elicited bactericidal effects of complement. Mammary epithelial cells were more sensitive to activation in response to rough MPEC strains which might explain earlier elimination and reduced fitness of these strains in the mammary gland. Previous studies suggested that increased coverage of lipid A (TLR4 ligand) by longer (smooth) O-polysaccharides chains might preclude access of C5b-C9 to lethal sites on the cell surface and protect smooth bacteria against complement as well as decrease activation of complement and of host cells by TLR4 signaling [63]. Interestingly, few of the MPEC field strains in our collection were rough and complement-resistant. Such strains, probably expressing alternative complements-resistance mechanism [64], might be more common in other environments and might also be the etiology of severe disease in dairy animals.

In conclusion, our results further support previously published results describing the role of LPS O-polysaccharides in mammary virulence [15, 65] and underline the importance of complement activity for mammary protection against bacterial colonization and infection. Previous studies also indicated that complement activity in the milk of dairy cows is highly variable and linked to genetic susceptibility to mastitis [44, 66–69]. Together with our results, these studies support the notion that complement activity in milk might be a good target for breeding mastitis-resistant dairy animals [70].

## Supplementary information

**Additional file 1: List of primers used for quantitative RTPCR analysis.**

**Additional file 2: Systemic virulence of MPEC P4-NR and loss of virulence of the derived strain P4-96 in wax moth larvae**. Virulence of P4-NR in *Galleria mellonella* larvae (A) was demonstrated following increasing challenge dose of bacteria (10^3^–10^6^ CFUs) associated with decreasing survival rates (B). Survival rates of larvae were compared following challenge with 10^8^ CFUs of P4-NR, P4-96 and the avirulent control strain BW25113 demonstrating attenuation and loss of virulence of strain P4-96 (C). Virulence of strain P4-NR was further demonstrated by analyzing growth in challenged larvae using culture techniques (black data points in D; mean + SE) and intravital bioluminescence imaging (E). Attenuation of the derived strain P4-96 was further demonstrated by lack of growth in challenged larvae (black squares in D; mean + SE). For intravital imaging, larvae were challenged with strain P4-NR constitutively expressing luminescence reporter and images were captures every 15 min over 9 h (E) using IVIS Lumina Series III (PerkinElmer Inc., MA, USA) in live larvae. Photon counts in the larvae were detected by CCD camera digitizer and were converted to physical units of radiance in photons/s/cm^2^/steradian. Regions of interest (ROI) were drawn around each individual larva and quantified with living Image Software version 4.4 (Caliper LifeSciences, MA, USA). Mean radiance (+SE) of live P4-NR bacteria in live larvae is presented in graph E. Survival rates of wax moth larvae following bacterial challenge (C) were compared using the Log-rank (Mantel-Cox).

**Additional file 3: Most field MPEC strains smooth**. A collection of clinically and epidemiologically defined MPEC field strains was analyzed using the plate acriflavine agglutination test and 45/48 (94%) were smooth. The three boxed strains are MPEC 26, MPEC 27 and MPEC 30.

**Additional file 4: Rough MPEC field strains are better activators of inflammatory response in mammary epithelial cells.** Rough MPEC strains 26, 27 and 30 were isolated from the milk of Israeli dairy cows affected by clinical mastitis (see Additional file 2). Using lentivirus vector, mammary epithelial cells EPH4 were transduced with luciferase gene under the control of 5 copies of the NF-kappa-B response element. Transduced EPH4 cells were treated with the following *E. coli* strains at MOI = 1; P4 (blue line), P4-NR (black line), MPEC 26 (red line), MPEC 27 (yellow line) and MPEC 30 (green line). NF-kappa-B activation was measured via analysis of luciferase activity in the presence of 150 µg/mL D-luciferin (RLU; relative luminescence units) following infection with the above described bacterial strains using SpectraMax i3x multiple detection microplate reader (Molecular Devices, CA, USA). In all experiments, LPS and normal medium were used as positive and negative controls, respectively. Data points are mean for one experiment performed in triplicate and represent the results of the three similar experiments.

## Abbreviations

*E. coli*: *Escherichia coli*
MPEC: Mammary pathogenic *E. coli*
LPS: Lipopolysaccharides
MLP: Microbial lipoproteins
TLR2: Toll-like receptor 2
TLR4: toll-like receptor 4
MAMP: Microbe-associated molecular pattern
IMM: Intramammary
MAEC: Mammary alveolar epithelial cells
