# Supplementary material for: The role of O-polysaccharide chain and complement resistance of Escherichia coli in mammary virulence
Source: Vet Res. 2020 Jun 15;51:77. doi: 10.1186/s13567-020-00804-x (PMC7294653; doi:10.1186/s13567-020-00804-x)
Supplement: Supplementary file 1 — Additional file 1: List of primers used for quantitative RTPCR analysis. [file 13567_2020_804_MOESM1_ESM.docx]

**Additional Table 1:** List of primers used for quantitative RTPCR analysis.

| **Gene** |  | **Forward primers 5'-3'** | **Reverse primers 5'-3'** | **Product length (bp)** |
| --- | --- | --- | --- | --- |
| Interleukin-10 | IL-10 | TGGCCTTGTAGACACCTTGG | AGCTGAAGACCCTCAGGATG | 264 |
| The tumor necrosis factor alpha | TNF-a | CATCTTCTCAAAATTCGAGTGACAA | TGGGAGTAGACAAGGTACAACCC | 175 |
| Inducible nitric oxide synthase | iNOS | CAGCTGGGCTGTACAAACCTT | CATTGGAAGTGAAGCGTTTCG | 95 |
| Interleukin-1 beta | IL-1 b | GGTCAAAGGTTTGGAAGCAG | TGTGAAATGCCACCTTTTGA | 94 |
| Chemokine (C-X-C motif) ligand 1 | Cxcl1 / KC | CGCTCGCTTCTCTGTGCA | ATTTTCTGAACCAAGGGAGCT | 242 |
| Cxcl2 chemokine (C-X-C motif) ligand 2 | Cxcl2 / Mip2 | TGCCTGAAGACCCTGCCAAGG | GTTAGCCTTGCCTTTGTTCAG | 189 |
| lymphocyte antigen 6 complex, locus G | Ly6G | TGCCCCTTCTCTGATGGATT | TGCTCTTGACTTTGCTTCTGTGA | 79 |
| Heat Shock Protein 90 | HSP90 | ATGAGTTGGGCAATTTCTGC | GTCTGGGTATCGGAAAGCAA | 137 |
