# Supplementary figures and images for: The role of O-polysaccharide chain and complement resistance of Escherichia coli in mammary virulence
Source: Vet Res. 2020 Jun 15;51:77. doi: 10.1186/s13567-020-00804-x (PMC7294653; doi:10.1186/s13567-020-00804-x)

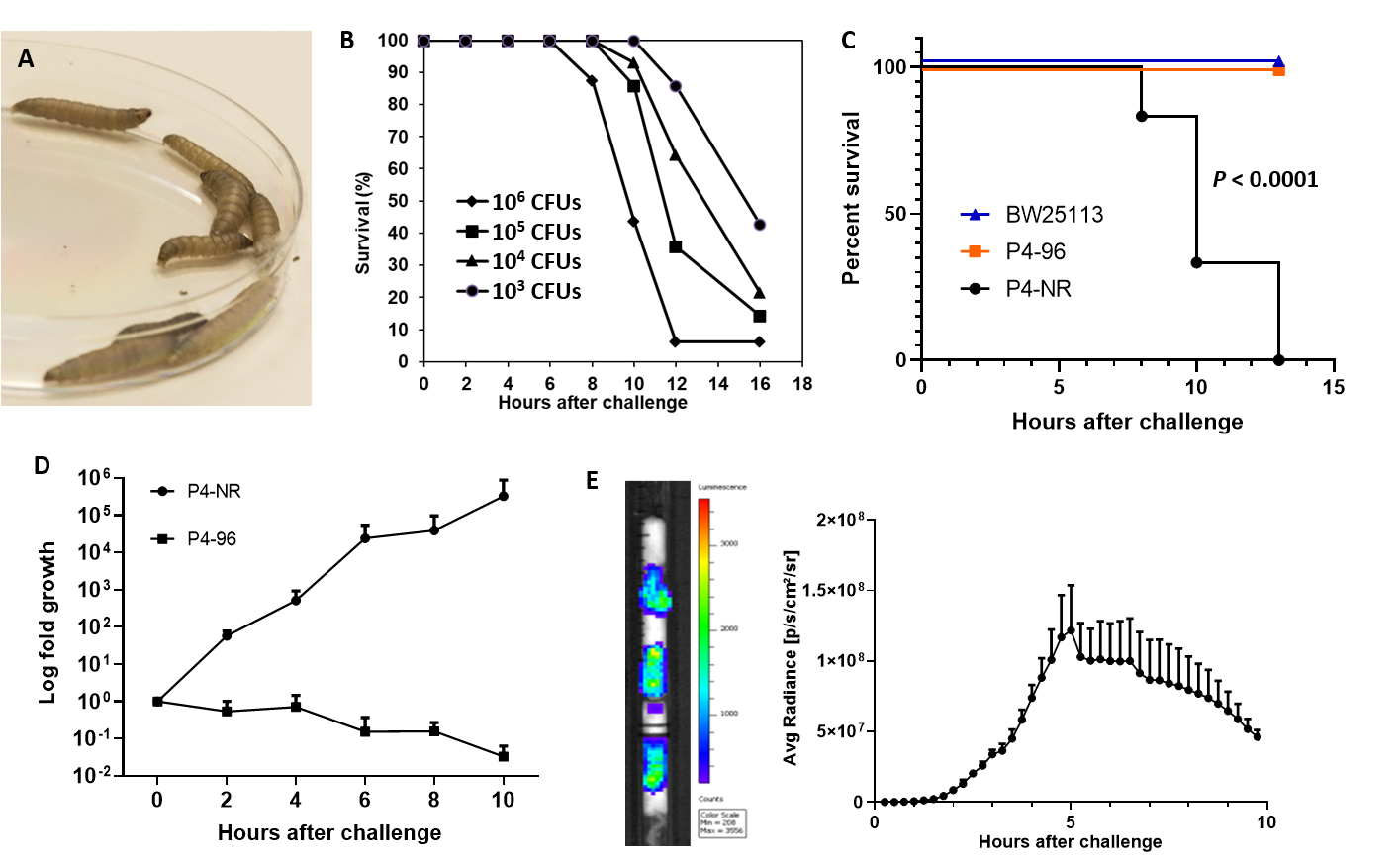

Supplement: Supplementary file 2 — Additional file 2: Systemic virulence of MPEC P4-NR and loss of virulence of the derived strain P4-96 in wax moth larvae. Virulence of P4-NR in Galleria mellonella larvae (A) was demonstrated following increasing challenge dose of bacteria (103–106 CFUs) associated with decreasing survival rates (B). Survival rates of larvae were compared following challenge with 108 CFUs of P4-NR, P4-96 and the avirulent control strain BW25113 demonstrating attenuation and loss of virulence of strain P4-96 (C). Virulence of strain P4-NR was further demonstrated by analyzing growth in challenged larvae using culture techniques (black data points in D; mean + SE) and intravital bioluminescence imaging (E). Attenuation of the derived strain P4-96 was further demonstrated by lack of growth in challenged larvae (black squares in D; mean + SE). For intravital imaging, larvae were challenged with strain P4-NR constitutively expressing luminescence reporter and images were captures every 15 min over 9 h (E) using IVIS Lumina Series III (PerkinElmer Inc., MA, USA) in live larvae. Photon counts in the larvae were detected by CCD camera digitizer and were converted to physical units of radiance in photons/s/cm2/steradian. Regions of interest (ROI) were drawn around each individual larva and quantified with living Image Software version 4.4 (Caliper LifeSciences, MA, USA). Mean radiance (+SE) of live P4-NR bacteria in live larvae is presented in graph E. Survival rates of wax moth larvae following bacterial challenge (C) were compared using the Log-rank (Mantel-Cox). [file 13567_2020_804_MOESM2_ESM.tif]

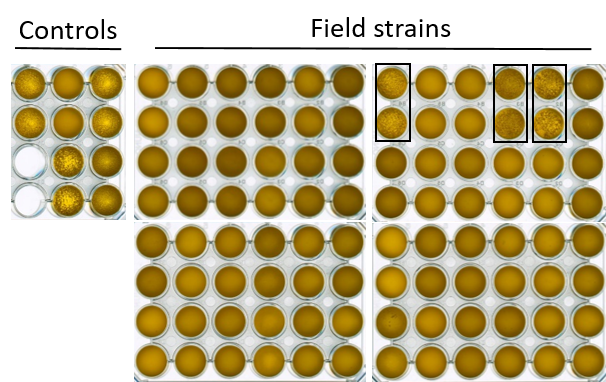

Supplement: Supplementary file 3 — Additional file 3: Most field MPEC strains smooth. A collection of clinically and epidemiologically defined MPEC field strains was analyzed using the plate acriflavine agglutination test and 45/48 (94%) were smooth. The three boxed strains are MPEC 26, MPEC 27 and MPEC 30. [file 13567_2020_804_MOESM3_ESM.tif]

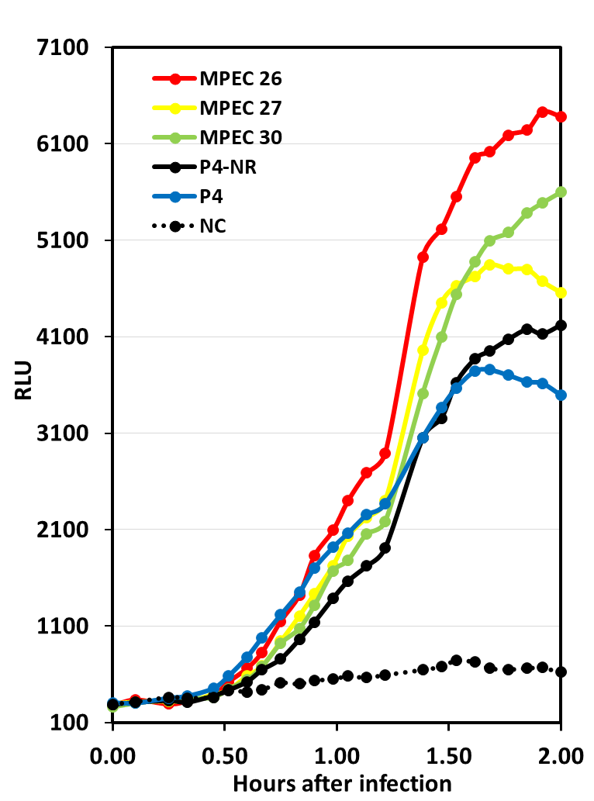

Supplement: Supplementary file 4 — Additional file 4: Rough MPEC field strains are better activators of inflammatory response in mammary epithelial cells. Rough MPEC strains 26, 27 and 30 were isolated from the milk of Israeli dairy cows affected by clinical mastitis (see Additional file 2). Using lentivirus vector, mammary epithelial cells EPH4 were transduced with luciferase gene under the control of 5 copies of the NF-kappa-B response element. Transduced EPH4 cells were treated with the following E. coli strains at MOI = 1; P4 (blue line), P4-NR (black line), MPEC 26 (red line), MPEC 27 (yellow line) and MPEC 30 (green line). NF-kappa-B activation was measured via analysis of luciferase activity in the presence of 150 µg/mL D-luciferin (RLU; relative luminescence units) following infection with the above described bacterial strains using SpectraMax i3x multiple detection microplate reader (Molecular Devices, CA, USA). In all experiments, LPS and normal medium were used as positive and negative controls, respectively. Data points are mean for one experiment performed in triplicate and represent the results of the three similar experiments. [file 13567_2020_804_MOESM4_ESM.tif]
